# The N-terminal Helix Controls the Transition between the Soluble and Amyloid States of an FF Domain

**DOI:** 10.1371/journal.pone.0058297

**Published:** 2013-03-07

**Authors:** Virginia Castillo, Fabrizio Chiti, Salvador Ventura

**Affiliations:** 1 Institut de Biotecnologia i de Biomedicina, Universitat Autònoma de Barcelona, Bellaterra, Spain; 2 Departament de Bioquímica i Biologia Molecular, Facultat de Biociències, Universitat Autònoma de Barcelona, Bellaterra, Spain; 3 Dipartimento di Scienze Biochimiche, Università di Firenze, Firenze, Italy; National Institute for Agricultural Research, France

## Abstract

**Background:**

Protein aggregation is linked to the onset of an increasing number of human nonneuropathic (either localized or systemic) and neurodegenerative disorders. In particular, misfolding of native α-helical structures and their self-assembly into nonnative intermolecular β-sheets has been proposed to trigger amyloid fibril formation in Alzheimer’s and Parkinson’s diseases.

**Methods:**

Here, we use a battery of biophysical techniques to elucidate the conformational conversion of native α-helices into amyloid fibrils using an all-α FF domain as a model system.

**Results:**

We show that under mild denaturing conditions at low pH this FF domain self-assembles into amyloid fibrils. Theoretical and experimental dissection of the secondary structure elements in this domain indicates that the helix 1 at the N-terminus has both the highest α-helical and amyloid propensities, controlling the transition between soluble and aggregated states of the protein.

**Conclusions:**

The data illustrates the overlap between the propensity to form native α-helices and amyloid structures in protein segments.

**Significance:**

The results presented contribute to explain why proteins cannot avoid the presence of aggregation-prone regions and indeed use stable α-helices as a strategy to neutralize such potentially deleterious stretches.

## Introduction

The function of a large majority of polypeptides depends on the attainment of a globular, compact and specific three-dimensional structure after their synthesis at the ribosomes [1]. Only properly folded globular proteins can interact specifically with their molecular targets [2]. The protein quality machinery works to minimize the accumulation of misfolded species, not only because they are not functional, but also because these conformers often display an intrinsic propensity to self-assemble into toxic aggregates, provoking the impairment of essential cellular processes. Accordingly, protein misfolding and aggregation lie behind an increasing number of human diseases that include highly debilitating disorders like Alzheimer’s or Parkinson’s disease [3]. Despite the polypeptides causing these pathologies are not related in terms of sequence or structure, in many cases their aggregation leads to the formation of amyloid fibrils, all sharing a common cross-β motif [4]. Moreover, the adoption of amyloid-like conformations is not restricted to disease-linked proteins and might constitute a generic property of polypeptide chains [5], [6], [7], likely because the non-covalent contacts that stabilize native structures resemble those leading to the formation of amyloids [8].

It was initially thought that, for globular proteins, amyloid fibril formation involved the docking of monomeric partially folded states, which display pre-existent β-sheet structure. Nevertheless, it was early shown that all-α proteins can also be induced to form amyloids under strongly destabilizing conditions. In particular, Dobson and co-workers demonstrated that in the case of apomyoglobin, amyloid fibril formation correlates with environments in which the protein backbone is unfolded, rather than with conditions that may allow population of partially structured states enriched in β-sheet conformations [9], [10]. Destabilization of apomyoglobin by mutation of two highly conserved Trp residues to Phe also results in the formation of amyloid fibrils, under conditions close to physiological [11]. For this double mutant, solution conditions that promote the population of the native α-helical secondary structure abolish the polymerization of the protein [11], illustrating a competition between folding and aggregation. In the present work we use the FF domain to provide further insights into the mechanism of amyloid fibril formation by α-helical proteins.

FF domains are small protein-protein interaction modules consisting of ∼50–70 residues often organized in tandem arrays and characterized by the presence of two conserved Phe residues at the N- and C-termini [12]. The three-dimensional structures of several FF domains have been solved, showing that this fold consists of three α-helices arranged as an orthogonal bundle with a 3_10_ helix in the loop connecting the second and the third helix [13], [14], [15]. They are involved in RNA splicing, signal transduction and transcription processes [16], [17]. These domains are present in a variety of eukaryotic nuclear transcription and splicing factors as well as in p190RhoGTPase-related proteins and their sequences are well conserved from yeast to humans [12]. However, the sequences of different FF domains are highly divergent. The loops connecting the different α-helical regions display the highest sequential variability, both in length and amino acidic composition. The main structural difference between divergent FF domains is the orientation, and sequence, of the second helix, which has been proposed as the structural element responsible for ligand specificity.

The folding process of the FF domain from human HYPA/FBP11 (HYPA/FBP11-FF) has being characterized in detail [18], [19], [20], [21]. This domain is receiving considerable attention, especially because it forms early in the folding reaction an on-pathway, short-lived and low populated intermediate state whose structure has been solved at atomic resolution combining NMR relaxation dispersion methods and computational techniques, providing thus a molecular description of a transient folding intermediate with unprecedented detail [22], [23], [24], [25].

**Figure 1:**
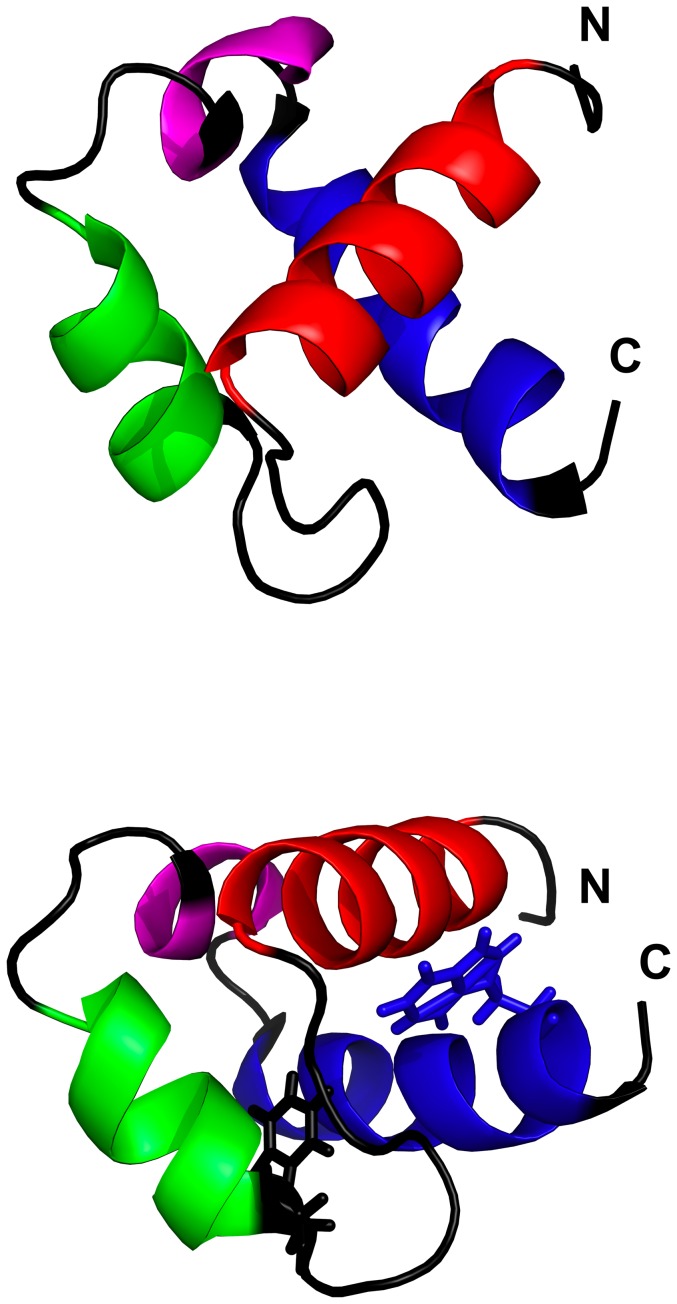
Structure of the URN1-FF domain. At the top, a ribbon representation showing the α-helices in different colors: α-helix 1 in red; α-helix 2 in green, helix 3_10_ in magenta; and α-helix 3 in blue. At the bottom, side chains of Trp 27 and Trp 56 are represented in black and blue, respectively. The N- and C-termini are indicated. The Protein Data Bank accession code for the structure is 2JUC. This figure was prepared with PyMOL.

The aggregation properties of FF domains have not been characterized yet. Here, we address this issue using the yeast URN1 FF domain (URN1-FF) as a model system. This is the only FF domain of the yeast URN1 protein, it consists of 59 residues and adopts a canonical α1−α2−3_10_−α3 FF fold (Figure 1). Relative to HYPA/FBP11-FF, the three α-helices are shorter in the yeast domain. Also, helices 1 and 3 are closer and more orthogonal in URN1-FF. We show here that URN1-FF forms amyloid fibrils at low pH. Using a battery of biophysical techniques to study the conformational, thermodynamic and kinetic properties of soluble URN1-FF, the morphological, structural and tinctorial properties of its aggregates as well as dissecting this domain into its individual secondary structure elements, we demonstrate that helix 1 at the N-terminus plays a key role in controlling the conformation and solubility of this small all-α protein.

## Materials and Methods

### Protein Expression and Purification

The URN1-FF domain corresponds to residues 212–266 of yeast URN1 and was cloned into a pETM-30 vector as an N-terminal fusion protein with a His tag followed by GST and a TEV protease cleavage site [15]. For protein production, the plasmid was transformed in BL21 (DE3) cells and after growing to 0.6 optical density they were induced with 1 mM IPTG at 298 K overnight. As a first step of purification, a His-tag column was used to isolate the GST-fused protein. Subsequently, a TEV cleavage was performed and a final gel filtration HiLoad™ Superdex™ 75 prepgrade (GE healthcare Life Sciences) was used to remove the GST protein. The sample was dialyzed against water and lyophilized. The purity of the samples was checked by SDS-PAGE and MALDI-TOFF mass spectroscopy. Protein concentration was determined by UV absorption using a ε value of 1.948 mg^−1^ ml cm^−1^.

### Sample Preparation for URN1-FF and Synthetic Peptides Conformational Assays

Lyophilized URN1-FF protein was dissolved at 20 µM using the following solutions: 50 mM potassium chloride at pH 1.5 and 1.75; 50 mM glycine at pH 2.0, 2.25, 2.5, 2.75 and 3.0; 50 mM sodium acetate at pH 3.5, 4.0, 5.0, 5.5 and 5.7; and 50 mM MES at pH 6.0 and 6.5. Protein solutions were filtered through a 0.22 µm filter and immediately analyzed at 298 K by Tryptophan intrinsic fluorescence, far-UV CD, static light scattering and ANS binding. Synthetic peptides were dissolved in 100 mM glycine at pH 2.5 and sonicated during 10 minutes. In all the cases the final concentration was 100 µM and different amounts of TFE were added between 0 and 25% (v/v).

### Sample Preparation for URN1-FF and Synthetic Peptides Aggregation Assays

Lyophilized URN1-FF protein was dissolved at 140 µM in different buffers and filtered through a 0.22 µm filter. Five different pH conditions were chosen to check aggregation: 100 mM glycine at pH 2.0, 2.5 and 3.0; and 100 mM sodium acetate at pH 4.0 and 5.7. Lyophilized synthetic peptides were prepared at 500 µM in 100 mM glycine at pH 2.5 in the presence of 0, 15 and 25% (v/v) of TFE and sonicated during 10 minutes. In all cases, the samples were incubated under agitation at 400 rpm and 310 K during 7 days.

### Aggregation Kinetics

URN1-FF protein was prepared at 140 µM in 100 mM glycine at pH 2.5 in the presence of 25 µM of ThT. Immediately after equilibrating the sample at 310 K during 5 minutes, ThT intrinsic fluorescence and light scattering intensity were measured every 5 minutes during 2000 minutes. The sample was excited at 440 nm and emission was recorded at 475 nm for ThT. For light scattering, the excitation and emission wavelengths were 340 and 350 nm, respectively. Slit widths of 5 nm were used for both excitation and emission in a CARY-100 Varian spectrophotometer.

### Electron Microscopy

Incubated samples were diluted tenfold with water and 10 µl were placed on carbon-coated copper grids and left for 5 min. The grids were then washed with distillated water and stained with 2% (w/v) uranyl acetate for 1 min. The analysis was done using a HITACHI H-7000 transmission electron microscope operating at an accelerating voltage of 75 kV. For diameter determination 173 and 108 different fibrils were analyzed at pH 2.0 and 2.5, respectively.

### Binding to Amyloid Dyes

URN1-FF aggregates were diluted at 10 µM in phosphate buffer pH 7.5 containing 25 µM of ThT. Synthetic peptides were studied at 40 µM with the same amount of ThT. ThT was excited at 440 nm and fluorescence emission was recorded between 460 and 600 nm, using excitation and emission slit widths of 10 nm. Each trace was the average of 3 accumulated spectra at 298 K in a CARY-100 Varian spectrophotometer.

To study the binding to CR, 30 µl of aggregated URN1-FF were mixed with 220 µl of CR (20 µM) in 5 mM phosphate, 150 mM NaCl pH 7.4 buffer at 298 K. After 5 minutes of equilibration, optical absorption spectra were acquired from 400 to 700 nm and accumulated for 3 times with a Jasco V-630 spectrophotometer (Tokyo, Japan). Solutions containing only protein and only CR were analyzed to eliminate the protein scattering and dye contribution.

### ANS Binding Assay

Aggregated samples were diluted at 10 µM in phosphate buffer pH 7.5 containing 25 µM of ANS. To study soluble URN1-FF species, samples were prepared at 20 µM containing 25 µM of ANS and analyzed immediately. The excitation wavelength was 370 nm and the emission spectra was recorded between 400 and 600 nm, using excitation and emission slit widths of 5 and 10 nm, respectively. Three spectra were accumulated after 5 minutes of equilibration at 298 K in a CARY-100 Varian spectrophotometer.

### NMR Spectroscopy

Lyophilized protein was dissolved at 35 µM in water using a 9∶1 H_2_O/D_2_O ratio, and adjusted at pH 2.5 and pH 5.7. One-dimensional NMR spectra were acquired at 298 K on a Bruker AVANCE 600-MHz spectrometer using solvent suppression WATERGATE techniques.

### Circular Dichroism, Intrinsic Tryptophan Fluorescence and Static Light Scattering

Monomeric and aggregated URN1-FF species were prepared at 20 µM and measured immediately. Far-UV CD spectra were measured in a Jasco-710 spectropolarimeter thermostated at 298 K. Spectra were recorded from 260 to 200 nm, at 0.2 nm intervals, 1 nm bandwidth, and a scan speed of 50 nm/min. Twenty accumulations were averaged for each spectrum. Both monomeric and aggregated synthetic peptides were diluted to a final concentration of 100 µM. Far-UV CD spectra were recorded in a Jasco J-810 spectropolarimeter (Tokyo, Japan) thermostated at 298 K, using the same conditions described above.

Tryptophan intrinsic fluorescence was measured at 298 K in a Cary-100 Varian spectrofluorometer using an excitation wavelength of 280 nm and recording the emission from 300 to 400 nm. Three averaged spectra were acquired and slit widths were typically 5 nm for excitation and emission. Soluble URN1-FF samples were measured in a PerkinElmer Life Sciences LS-55 fluorimeter (Wellesley, Massachusetts) equipped with a thermostated cell compartment using the same parameters described above. The represented values are the integral of the fluorescence between 300 and 400 nm.

Static light scattering was recorded in a Jasco V-630 spectrophotometer (Tokyo, Japan) at 298 K. Two accumulative spectra were registered between 360 and 240 nm.

### Thermal Denaturation

URN1-FF samples were dissolved at 20 µM and four different pH conditions (2.0, 2.5, 3.0 and 5.7). Thermal stabilities were studied by intrinsic fluorescence intensity and far-UV CD. The samples were excited at 280 nm and the emission was recorded at 360 nm, using slit widths of 5 nm for excitation and emission in a CARY-100 Varian spectrophotometer. The emission was registered each 0.25 K with a heating rate of 0.5 K/min. The molar ellipticity at 222 nm was recorded each 0.2 K with a heating rate of 0.5 K/min in a Jasco-710 spectropolarimeter.

### Equilibrium Stability Measurements

Lyophilized protein was dissolved at 20 µM in different pH buffers and molarities of urea, from 0 to 9 M and left to equilibrate for at least 3 h. The samples were equilibrated during 10 minutes at 310 K and analyzed by intrinsic fluorescence intensity and far-UV CD. The excitation and emission wavelengths were 280 and 360 nm, respectively, using a CARY-100 Varian spectrophotometer. Unfolding was also followed by far-UV CD at 222 nm in a Jasco-710 spectropolarimeter.

### Analysis of the Chemical Denaturation Curves

In chemical denaturations, experimental data were fitted using the Kaleidagraph version 4.0 (Synergy Software) to equation 1 assuming two-state unfolding with 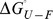 equal to equation 2:
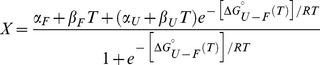
(1)

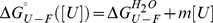
(2)



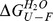
 is the stability of the protein in the absence of denaturant. *[U]* is the denaturant concentration and *m* is the urea concentration at the midpoint of the curve. Fraction folded at a given urea concentration was determined by using *X*, *α_F_*, *β_F_*, *α_U_*, and *β_U_*. *X* is the ellipticity at 222 nm or fluorescence intensity at 360 nm; *α_F_*, *β_F_*, *α_U_*, and *β_U_* are parameters that define ellipticity or fluorescence intensity of the folded (*F*) and unfolded states (*U*). *α_F_* and *α_U_* are the axis intercepts and *β_F_* and *β_U_* are the slopes of the pre- and post-transition baselines. *T* is temperature, and *R* is the gas constant.

### Folding Kinetics

Kinetics of unfolding and refolding reactions at 298 K and 310 K were followed in a Bio-Logic SFM-3 stopped-flow instrument using excitation at 280 nm and a 320 nm fluorescence cut-off filter. Unfolding reaction was promoted by dilution of the protein in buffer with appropriate volumes of the same buffer containing 9.5 M urea. For folding reactions, appropriate volumes of buffer were added to an initial protein solution containing 9.5 M urea. To determine the unfolding rates at 8 M of urea, protein and urea solutions were prepared at different pH and unfolding kinetics were recorded at 310 K in a Bio-Logic SFM-3 device.

Observed rate constants were fitted to the equation describing folding of a two-state protein, using the Kaleidagraph version 4.0 (Synergy Software). To determine the free energy, m and C_M_ values we used the following equations:
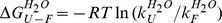


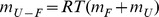


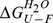
where k_F_ and k_U_ are the rate constants for folding and unfolding, respectively, and the m_F_ and m_U_ values correspond to the slopes of the respective folding and unfolding regions.

### Prediction of the Aggregation-prone Regions and α-helical Propensity of URN1-FF Domain

The primary sequence of URN1-FF was used as input to predict the regions prone to aggregation. We used five different algorithms: WALTZ [26], AGGRESCAN [27], BETASCAN [28], FOLDAMYLOID [29], AMYLPRED [30], ZYGGREGATOR [31] and PASTA [32]. The tendency of URN1-FF to adopt α-helical structure was predicted using AGADIR (http://agadir.crg.es/).

### Pepsin Digestion

Limited proteolysis was carried out using pepsin in 50 mM glycine buffer pH 2.5 and 35 µM of URN1-FF protein. The digestion was performed at 310 K containing an E/S ratio of 1∶200 (by weight) and the reactions were quenched after 0.5, 1 and 5 min by adding an appropriate volume of ammonia 2%. The proteolytic mixtures were analyzed by Tricine-SDS/12% PAGE and by MALDI-TOFF MS using an Ultraflex spectrometer (Bruker) operating in linear mode under 20 kV. Samples were prepared by mixing equal volumes of the protein solution and matrix solution (10 mg/ml of sinapic acid dissolved in aqueous 30% acetonitrile with 0.1% TFA) and using the dried droplet method. A mixture of proteins from Bruker (protein calibration standard I; mass range = 3–25 kDa) was used as external mass calibration standard. Peptide fragments were identified by mass fingerprinting analysis.

## Results

### pH Dependence of URN1-FF Conformational Properties

**Figure 2:**
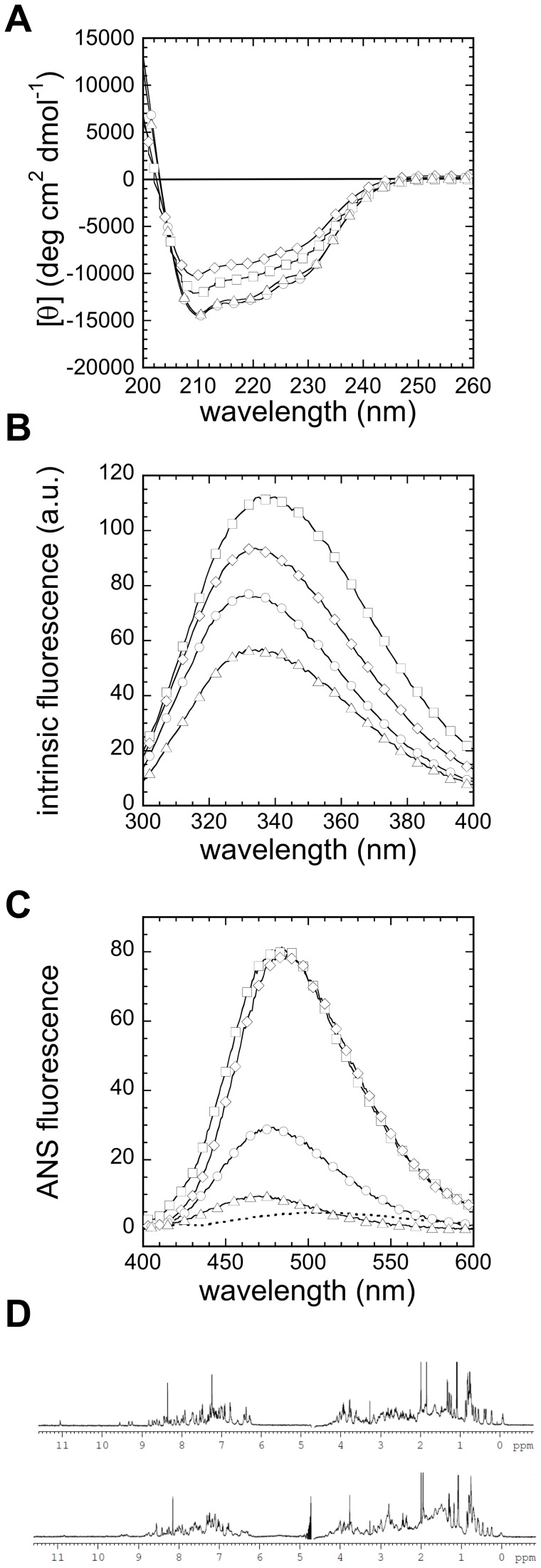
pH dependence of URN1-FF conformational properties. Protein samples were prepared at 20 µM and were immediately measured by (**a**) far-UV CD, (**b**) tryptophan intrinsic fluorescence and (**c**) ANS fluorescence at 298 K. The fluorescence emission spectrum of ANS in the absence of protein is represented as a dotted line. URN1-FF species were at pH 2.0 (squares), pH 2.5 (diamonds), pH 3.0 (circles), and pH 5.7 (triangles). (**d**) One-dimensional NMR (^1^H-NMR) spectra of URN1-FF were recorded at 298 K and 600 MHz, using a protein concentration of 35 µM. Two different spectra were collected, at pH 5.7 (above) and pH 2.5 (below).

In contrast to HYPA/FBP11-FF, which displays a basic pI of 9.9, the pI of URN1-FF is 4.4. We studied the conformational properties of URN1-FF over the pH range 2.0–5.7 and at 298 K by far-UV circular dichroism (CD), intrinsic fluorescence and 1-anilinonaphtalene-8-sulfonate (ANS) binding at 20 µM protein concentration (Figure 2).

The far-UV CD of URN1-FF at pH 5.7 is similar to that reported for HYPA/FBP11-FF in the same conditions and, in agreement with its solution NMR structure, it corresponds to that of a canonical α-helical protein, displaying the characteristic minima at 210 and 222 nm (Figure 2A). Analysis of the CD spectra with the K2D3 algorithm predicts a secondary structure content consisting of 95% of α-helix and 5% of random coil without any significant β-sheet component. The far-UV CD spectra of URN1-FF solutions at different acidic pHs (2.0, 2.5 and 3.0) all display an α-helical spectra similar to that under native conditions at pH 5.7, despite exhibiting reduced ellipticity at pH 2.0 (Figure 2A), suggesting that this domain retains a significant amount of their native secondary structure at low pH. K2D3 predicted an α-helix content of 95%, 95% and 93% at pH 3.0, 2.5 and 2.0, respectively, without any significant contributions of β-sheet signal. In contrast to the other conditions, the protein solution becomes cloudy at pH 4.0. This was probably because this pH is close to the URN1-FF pI, resulting in isoelectric protein precipitation. Therefore this condition was not further analyzed.

URN1-FF contains two buried Trp residues at positions 27 and 56. We monitored the pH-induced changes in the tertiary structure of this domain following the variation in Trp emission. A progressive increase in fluorescence intensity and a red shift of the maximum emission wavelength was observed as pH decreases, indicating an increase in the solvent exposure of these aromatic residues (Figure 2B). Importantly, at the lowest pH value tested here the emission maximum was shifted to ∼335–340 nm, indicating that the Trp residues are not fully solvent-exposed. This suggested opening of the native conformation with the concomitant partial exposure of hydrophobic clusters at low pH. To test this possibility further, we analyzed the binding of URN1-FF to ANS at the different pHs. At all pH values the protein was found to increase the fluorescence of ANS and cause a blue-shift of its emission maximum. However, a dramatic increase in ANS fluorescence was observed at pH 2.0 and 2.5, with intermediate and low ANS fluorescence increases at pH 3.0 and 5.7, respectively (Figure 2C).

The structural features of the URN1-FF at pH 2.5 and 5.7 were also evaluated by NMR spectroscopy. The one-dimensional NMR (^1^H NMR) spectrum of the protein at pH 5.7 displayed a wide dispersion of resonances at both low (amide and aromatic region) and high (methyl region) fields, with good peak sharpness, characteristic of folded molecules (Figure 2D). At pH 2.5, certain peak collapse was observed. However, the signal dispersion is indicative of the retention of a significant number of URN1-FF intramolecular contacts at this pH.

Overall, the structural features of URN1-FF at acidic pH and 298 K are compatible with the population of a molten globule-like structure in these conditions. To assess the stability of the detected URN1-FF species under these conditions of temperature and protein concentration, we monitored the evolution of their conformational properties in a wide range of pH, from 1.5 to 6.5 along time. In the 2.0–6.5 pH range no changes in tertiary structure, as monitored by Trp fluorescence, and secondary structure, as monitored by CD, were detectable upon 24 h incubation (Figure S1). Accordingly, with the exception of pH 4.0, no aggregation signs were visible by light scattering in this time period and pH range (Figure S1), indicating that the conformational ensembles are at least metastable.

### pH Dependence of URN1-FF Thermal and Chemical Stability

**Figure 3:**
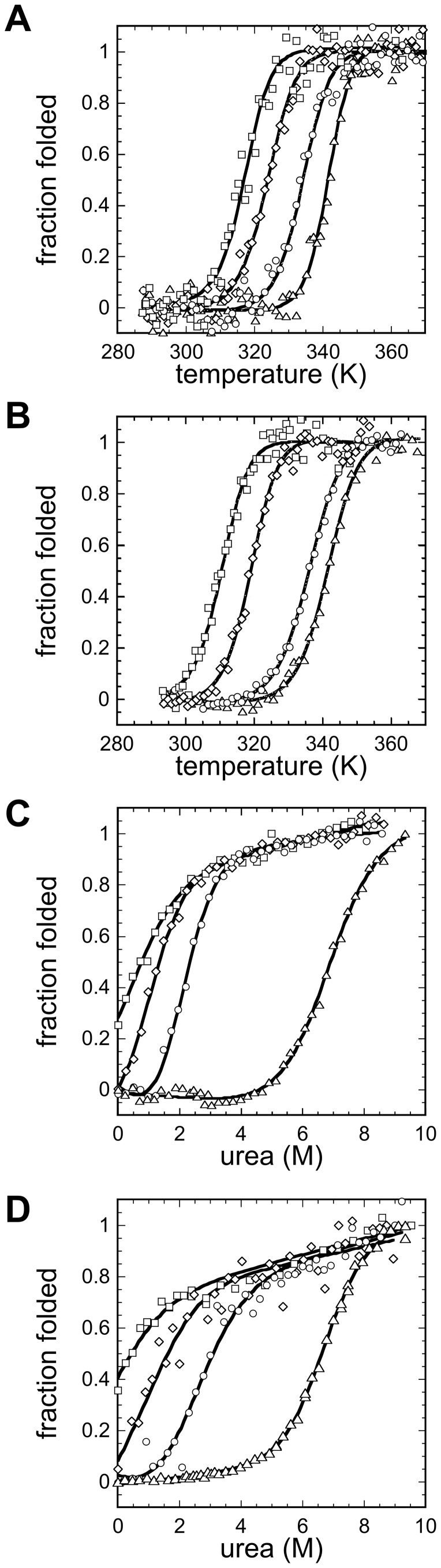
Thermal and Chemical stabilities of URN1-FF at different pHs. Thermal stabilities were studied monitoring the changes by (**a**) far-UV CD at 222 nm and by (**b**) intrinsic fluorescence at 350 nm. Equilibrium urea unfolding curves at 310 K were followed by (**c**) far-UV CD at 222 nm and by (**d**) tryptophan intrinsic fluorescence at 350 nm. Protein samples are represented at pH 2.0 by squares; pH 2.5, diamonds; pH 3.0, circles; and pH 5.7, triangles.

The thermal stability of URN1-FF under native conditions (pH 5.7) was analyzed following the changes in the far-UV CD spectra at 222 nm and in intrinsic fluorescence at 350 nm (Figure 3A and 3B). The two probes reported essentially identical thermal transitions, with a melting temperature T_m_ of 340.9±0.3 and 341.4±0.1 K, by far-UV CD and intrinsic fluorescence, respectively (Table 1). We then addressed the dependence of the thermal stability of the domain on the pH. At all assayed pHs, cooperative transitions were observed. Nevertheless, the thermal stability of URN1-FF is drastically affected by the pH. The T_m_ decreases with decreasing pH and is reduced by ∼30 K from pH 5.7 to 2.0 (Table 1). A good agreement between the T_m_ calculated from CD and fluorescence data is observed at all pHs.

**Table 1:** Melting temperatures of URN1-FF at different pHs.

	T_m_ (K)	
	CD^1^	Intrinsic fluorescence^2^
pH 2.0	313.5±1.3	310.9±0.3
pH 2.5	321.5±0.1	318.9±0.1
pH 3.0	332.8±0.1	335.8±0.1
pH 5.7	340.9±0.3	341.4±0.1

1 Changes in molar ellipticity were monitored at 222 nm. ^2^ Changes in intrinsic fluorescence were monitored at 350 nm.

To analyze differences in the stability of the different URN1-FF conformations, we studied the resistance of the protein at different pH values against chemical denaturation with urea at 310 K by monitoring the changes in molar ellipticity at 222 nm and in Trp fluorescence intensity at 350 nm (Figure 3C and 3D). We selected 310 K because at this temperature the percentage of folded URN1-FF strongly depends on the pH. As it will be seen in the next sections, this will allow us to correlate the degrees of native structure and aggregation. At pH 5.7 the FF domain unfolded in a cooperative, two-state process. Accordingly, the thermodynamic values obtained from fluorescence and CD measurements were similar (Table 2). 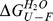 of 5.33±0.25 kcal/mol and 5.94±0.18 kcal/mol were calculated by far-UV CD and intrinsic fluorescence, respectively. A [urea]_50%_ of 6.75 M was obtained for both probes. This domain is significantly more stable than the structurally homologous human HYPA/FBP11 FF domain, which has a 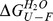 of 3.6 kcal/mol and a [urea]_50%_ of 3.1 M at the same pH.

**Table 2:** Thermodynamic characterization of URN1-FF at different pHs.

	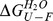 1 (kcal mol^−1^)		m^2^ (kcal mol^−1^ M^−1^)		[urea]_50%_ 3 (M)	
	CD^4^	Intrinsicfluorescence^5^	CD	Intrinsic fluorescence	CD	Intrinsic fluorescence
pH 2.0	0.36±0.04	0.051±0.08	0.83±0.04	0.4±0.1	0.4±0.1	0.2±0.8
pH 2.5	1.4±0.1	1.2±0.3	1.0±0.2	0.7±0.5	1.4±0.4	1.6±0.9
pH 3.0	2.2±0.3	1.8±0.6	1.1±0.1	0.8±0.1	2.0±0.3	2.3±0.9
pH 5.7	5.3±0.3	5.9±0.2	0.79±0.04	0.88±0.03	6.8±0.6	6.8±0.3
FBP11^6^	3.62±0.03	3.60±0.02	1.180±0.001	1.140±0.001	3.05±0.03	3.16±0.03

1 Gibbs energy of unfolding with urea determined from the equilibrium parameters.

2 Dependence of the Gibbs energy of unfolding with urea.

3 The urea concentration required to unfold 50% of the protein molecules.

4 Parameters obtained by following changes in the molar ellipticity at 222 nm.

5 Parameters obtained by following changes in the intrinsic fluorescence at 350 nm.

6 The values of the FF domain of HYPA/FBP11 were previously reported at 283 K [18], [19].

A dramatic decrease of URN1-FF thermodynamic stability was observed at lower pHs. At pH 3.0 and 2.5 the protein exhibited a complete cooperative transition. At pH 3.0, CD and fluorescence analysis rendered similar thermodynamic parameters showing that the protein is destabilized by ∼3.6 kcal/mol (Table 2). At pH 2.5, 5–10% of the protein population appears to be already unfolded at equilibrium in the absence of urea, being destabilized by ∼4.4 kcal/mol. As expected, at pH 2.0 the domain is partially unfolded and displays marginal stability since 310 K is close to the T_m_ at this pH.

### Folding and Unfolding Kinetics of the URN1-FF Domain

**Figure 4:**
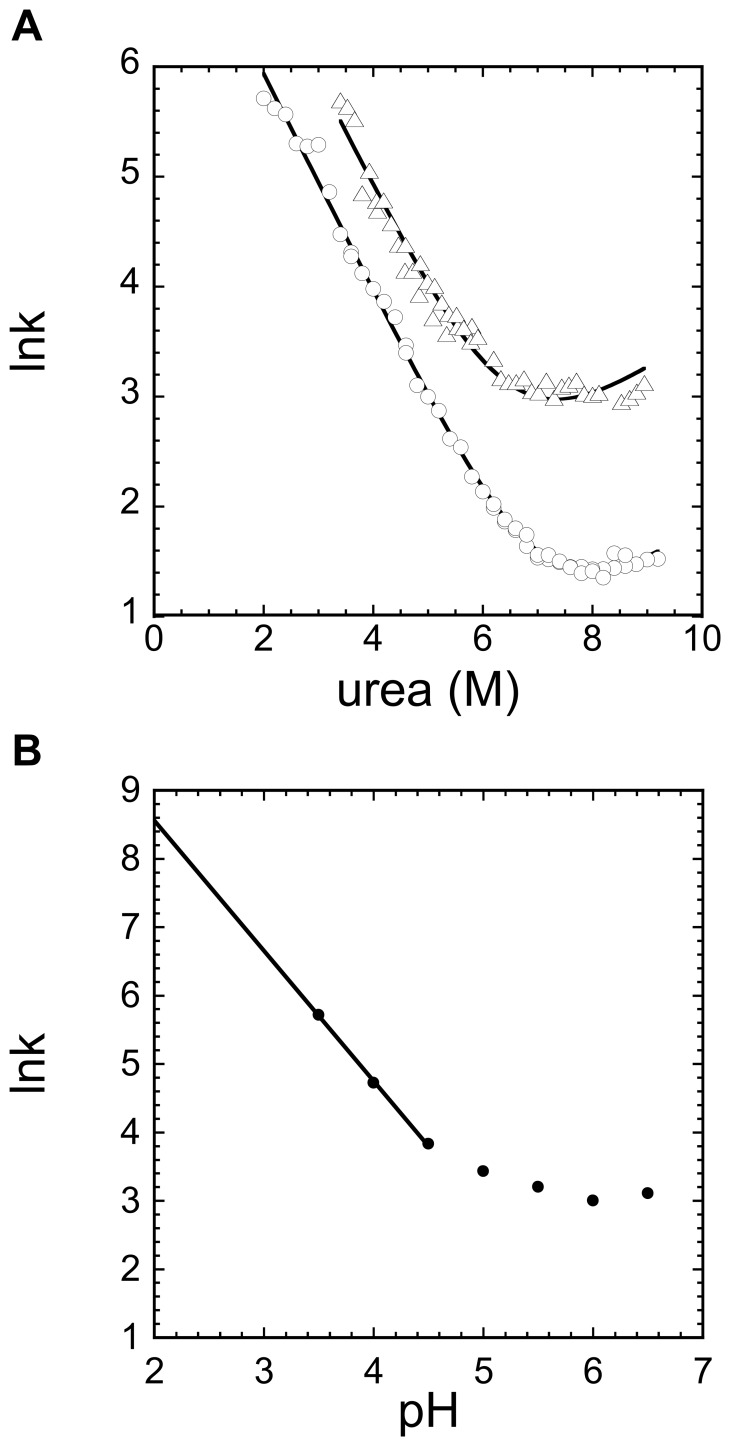
Unfolding and refolding kinetics of URN1-FF. (**a**) The kinetics of unfolding and refolding for URN1-FF at pH 5.7 were followed by Trp intrinsic fluorescence. Stopped-flow experiments were performed at 298 K (circles) and 310 K (triangles). (**b**) Unfolding rates in 8 M urea at pH ranging from 3.5 to 6.5 were recorded at 310 K. The rate constants were measured under conditions of apparent two-state folding.

The kinetics of folding and unfolding of the URN1-FF domain at pH 5.7 were determined by stopped-flow and fluorescence detection under a wide range of denaturant conditions at 298 and 310 K. In both cases, the folding and unfolding traces by fluorescence fit well into single exponential functions. Moreover, the chevron plots appear to be linear in the complete range of urea concentration studied (Figure 4A), indicating the lack of detectable intermediates, according to a two-state model. The rate constants for folding (k_F_) and unfolding (k_U_) and their dependence on denaturant concentration (RTm_F_ and RTm_U_) are shown in Table 3. The thermodynamic data at 310 K obtained from kinetic measurements are in good agreement with the equilibrium data at this temperature using chemical denaturation. Increasing the temperature from 298 to 310 K has a moderate effect on the thermodynamic stability of URN1-FF (∼0.3 kcal/mol), but it has an important impact on the folding and unfolding rates, which increase by 2.6 and 5.8 fold, respectively. The folding and unfolding kinetics of the HYPA/FBP11 domain have been characterized at 283 K. Unfortunately, URN1-FF could not be studied at this temperature since at the high concentrations required for unfolding reactions urea crystallizes, precluding direct comparison of kinetic traces. However, it is worth mentioning that even at 310 K the unfolding rate of URN1-FF is two fold slower than that of HYPA/FBP11-FF at 283 K, suggesting that the unfolding rate is a main contributor to the different thermodynamic stabilities exhibited by these two FF domains.

**Table 3:** Kinetic parameters for URN1-FF at pH 5.7.

	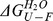 1(kcal mol^−1^)	m_U-F_ 2(kcal mol^−1^M^−1^)	C_m_ 3 (M)	k_F_ (s^−1^)	k_U_ (s^−1^)	RTm_U_(kcal mol^−1^ M^−1^)	RTm_F_(kcal mol^−1^ M^−1^)
298 K	5.57±0.28	0.78±0.04	7.13±0.55	2741±95	0.23±0.11	0.19±0.04	0.59±0.01
310 K	5.28±0.04	0.81±0.04	6.50±0.34	7022±221	1.33±0.06	0.20±0.04	0.61±0.01
FBP11^4^	3.64±0.05	1.16±0.02	3.14±0.07	2200±90	3.66±0.06	0.154±0.003	1.01±0.02

1 Gibbs energy of unfolding with urea determined from the kinetic parameters.

2 Dependence of the Gibbs energy of unfolding with urea.

3 The urea concentration required to unfold 50% of the protein molecules.

4 The values of the FF domain of HYPA/FBP11 were previously reported at 283 K [19].

The pH dependence of the unfolding rate at 8 M urea was measured at 310 K over the pH range 3.5–6.5 (Figure 4B). Below pH 3.5 the unfolding reaction was too fast to be monitored with our equipment. As it happens with HYPA/FBP11-FF, lowering the pH results in an increase in the k_U_ value for URN1-FF. The plot of ln (k_U_) versus pH allows the approximate determination of the unfolding rate constants at pH 2.5 and pH 2.0, 8 M urea and 310 K by extrapolation. Then, assuming that m_U_ is independent of pH, k_U_ values of ∼150 and ∼360 s^−1^ are calculated in the absence of urea at 310 K at pH 2.5 and 2.0, respectively. This indicates that the domain unfolding is very fast under these conditions.

### Effect of pH on the Aggregation of the URN1-FF Domain

**Figure 5:**
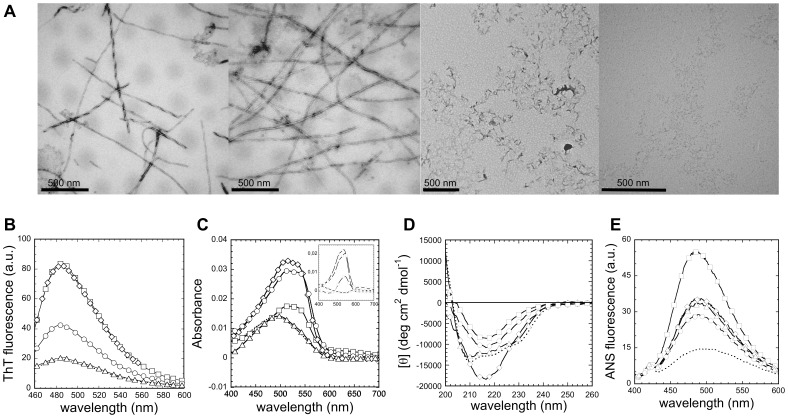
Morphological, structural and tinctorial properties of URN1-FF aggregates at different pHs. (**a**) Representative TEM images of URN1-FF aggregates at 140 µM under different pH conditions incubated at 310 K for one week. From left to right: pH 2.0, pH 2.5, pH 3.0 and pH 5.7. (**b**) Fluorescence emission spectra of ThT (25 µM) in the absence (dotted line) and in the presence of 10 µM of protein aggregates formed as in (a). (**c**) Absorption spectra of CR (16 µM) in the absence (dotted line) and in the presence of 17 µM of URN1-FF aggregates formed as in (a). The inset shows the difference spectra obtained by subtracting CR-phosphate buffer spectrum from protein in CR-phosphate buffer spectrum. (**d**) Far-UV CD spectra of native URN1-FF (dotted line) and aggregates, using a final concentration of 20 µM. The aggregates were formed as in (a). (**e**) Fluorescence emission spectra of ANS (25 µM) collected in the absence (dotted line) and in the presence of protein aggregates (10 µM) formed as in (a). In all the cases, protein aggregates at pH 2.0 are represented as squares; pH 2.5, diamonds; pH 3.0, circles; and pH 5.7, triangles.

Despite URN1-FF remains soluble at 20 µM at all pHs, with the exception of pH 4, it might aggregate at higher protein concentrations. To assess if the URN1-FF domain self-assembles into macromolecular structures in a pH dependent manner, the protein was therefore incubated at 140 µM and 310 K for 7 days over the pH range 2.0–5.7. The presence and morphology of protein aggregates was analyzed using Transmission Electron Microscopy (TEM) (Figure 5A). Fibrillar species were observed at both pH 2.0 and pH 2.5. The fibrils in both solutions were long and unbranched and consist of linear or twisted fibrils. Linear fibrils displayed a diameter of 7.01±1.14 and 7.48±1.5 nm at pH 2.0 and 2.5, respectively. The diameter of twisted fibrils was 14.29±4.33 nm at pH 2.0 and 16.13±4.13 nm at pH 2.5. The diameter of FF linear fibrils is consistent with that of the amyloids formed by pathological proteins, which usually ranges between 4 and 10 nm [33]. The dimensions of twisted fibrils suggest that they likely result from the association of two linear fibrils. At pH 3.0 only small protofibrillar-like aggregates were observable and at pH 5.7 the solution was essentially devoid of aggregates. Quantification of the amount of aggregated protein by sample fractionation using sedimentation at 100,000 *g* for 1 hour is consistent with the TEM analysis, showing that most of URN1-FF is aggregated at pH 2.0, 2.5 and 3.0, with 97%, 99% and 100% of the total protein being located in the insoluble fraction, respectively. On the contrary, 93% of the protein remained in the soluble fraction at pH 5.7. As expected, the protein solution became immediately cloudy at pH 4.0. Accordingly, large and amorphous aggregates were observed by TEM (Figure S2).

We used the amyloid-specific dyes thioflavin T (ThT) and Congo red (CR) to analyze if the aggregates detected in the different protein solutions exhibit amyloid-like features (Figure 5B and 5C). In agreement with their fibrillar appearance, the aggregates formed at pH 2.0 and 2.5 promoted the highest increase in ThT fluorescence emission, followed by the pH 3.0 protofibrillar assemblies; little ThT binding was observed at pH 5.7. With the exception of pH 5.7, incubation of URN1-FF at all other pHs promoted binding of the protein to CR resulting in a red-shift and an increase in the absorbance maximum of the dye, thus confirming the presence of amyloid-like aggregates formed in these conditions.

We then monitored the conformational properties of the URN1-FF aggregates incubated in the different acidic conditions using far-UV CD (Figure 5D). The protein incubated at 140 µM, pH 5.7, 310 K for 7 days exhibited essentially the same native spectrum as the freshly dissolved protein at 20 µM, displaying the characteristic minima at 210 and 222 nm (Figure 5D). In contrast, in FF domains incubated at pH 2.0, 2.5 and 3.0 the transition towards a β-sheet enriched conformation was evident.

We also monitored the exposure of hydrophobic clusters to the solvent in the aggregates attained at the different pHs with ANS (Figure 5E). The fibrils formed at pH 2.0 exhibited the highest ANS binding, suggesting conformational differences between these assemblies and the fibrils formed at pH 2.5, in line with their different binding to CR and β-sheet signal intensity in the CD spectra.

Overall, the data converge to indicate that the URN1-FF domain forms aggregates displaying different morphological and conformational properties depending on the pH. In particular, the aggregates at pH 2.0 and 2.5 have the morphology, structural and tinctorial characteristics typical of amyloid fibrils.

### The Aggregation Kinetics of the URN1-FF Domain at Low pH

**Figure 6:**
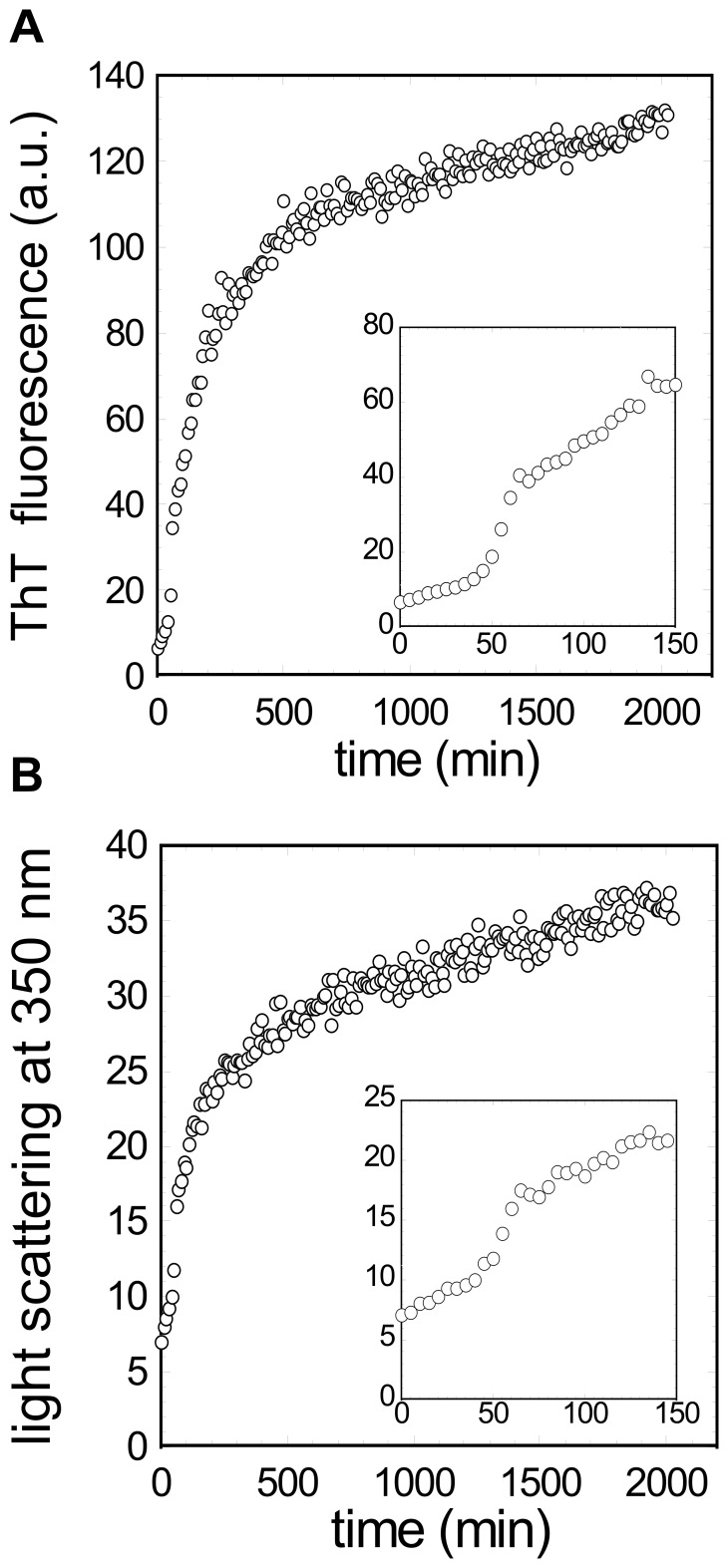
URN1-FF aggregation kinetics at pH 2.5. Change of (**a**) ThT fluorescence (25 µM) and (**b**) light scattering at 350 nm during aggregation of URN1-FF at 140 µM, pH 2.5 and 310 K. The insets show the same kinetic plots on an expanded time scale.

The time course of URN1-FF amyloid fibril formation at 140 µM, pH 2.5 and 310 K was monitored by light scattering and ThT fluorescence. The kinetics of amyloid fibril formation usually follows a sigmoidal curve that reflects a nucleation-dependent growth mechanism. The assembly of URN1-FF follows this kinetic scheme but exhibits a short lag phase (Figure 6 and Table S1). The kinetic traces, as well as the lag time and the elongation rate constant for the aggregation reaction, followed by ThT binding and light scattering were essentially identical, indicating that at pH 2.5, the formation of amyloid-like intermolecular interactions occurs rapidly in the aggregation process.

### Prediction of URN1-FF Sequence Segments with α-helical and Aggregation Propensities

**Figure 7:**
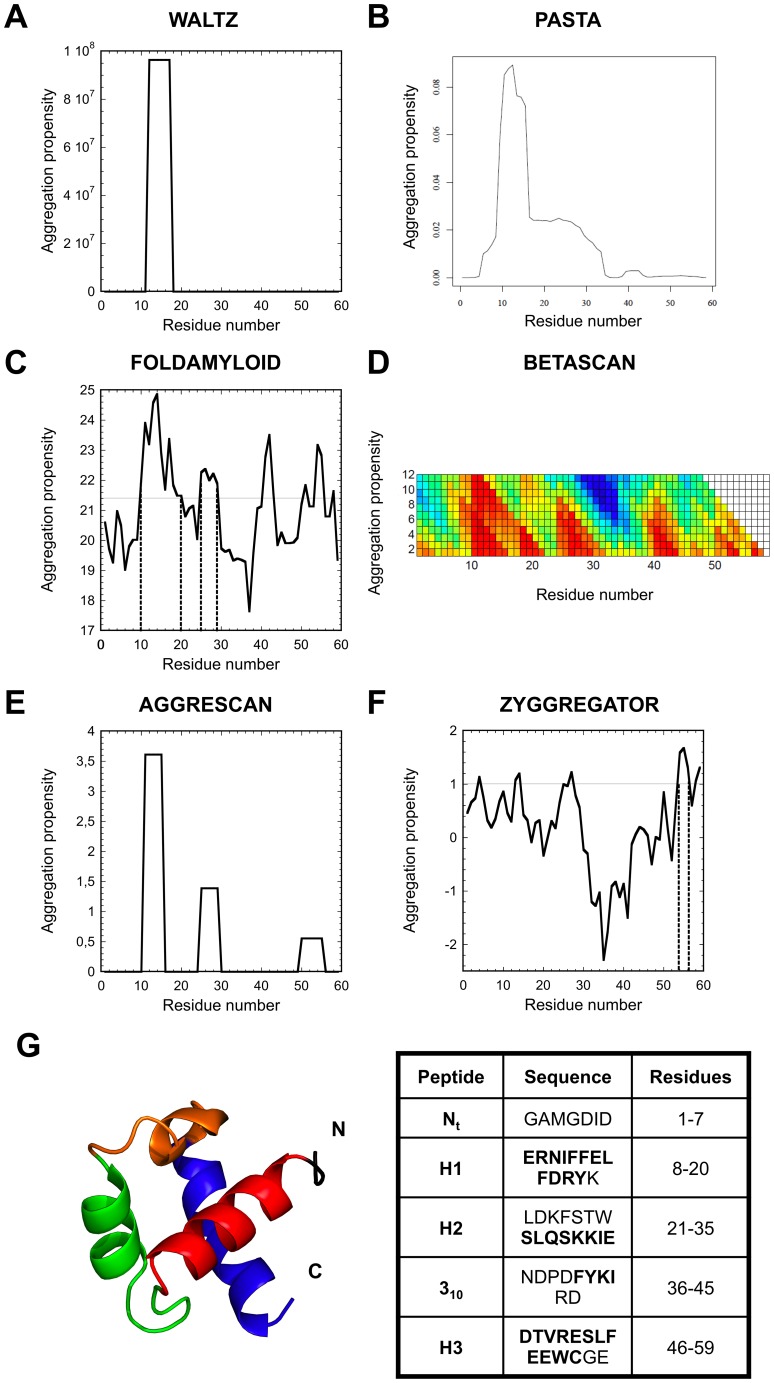
Prediction of aggregation-prone regions in URN1-FF and peptide design. Aggregation profiles were predicted using (**a**) WALTZ, (**b**) PASTA, (**c**) FOLDAMYLOID, (**d**) BETASCAN, (**e**) AGGRESCAN and (**f**) ZYGGREGATOR algorithms. (**g**) Ribbon diagram of the URN1-FF domain showing the designed peptides in different colours: N_t_ peptide in black, H1 peptide in red, H2 peptide in green, 3_10_ peptide in orange and H3 peptide in blue. The table contains the amino acid sequences corresponding to each peptide. The residues involved in the formation of α-helices in the native structure are shown in bold.

It is now accepted that specific and continuous sequence segments of a protein promote amyloid fibril formation and participate to the establishment of the β-core of the mature fibrils [34], [35]. Different computational methods have been developed to predict those sequential stretches [36], [37]. The evidence that at low pH URN1-FF forms amyloid fibrils indicates that this domain should posses at least one amyloidogenic region. We have used a number of the existing algorithms to identify the region of the URN1-FF sequence that most likely promote amyloid fibril formation of the protein. WALTZ detects the stretch spanning residues 12–17 in helix 1 at pH 2.5. Consistently, PASTA detects a single amyloidogenic region comprising residues 10–16. In addition to helix 1, BETASCAN, AMYLPRED and FOLDAMYLOID algorithms predict a second sequence comprising residues 25–29 at the end of loop 1 and beginning of helix 2. Finally, in addition to these two stretches, AGGRESCAN and ZYGGREGATOR suggest that residues 50–55 in helix 3 may also display significant aggregation propensity (Figure 7A–F).

The predicted α-helical propensity of the URN1-FF sequence was analyzed using AGADIR in the 2.4–5.8 pH range at 310 K. The global α-helical propensity of the protein is predicted to decrease with decreasing pH. At pH 5.7 the region of highest α-helical propensity corresponds to residues 6–21, including the complete helix 1 and the initial part of loop 1, the rest of residues displaying low intrinsic α-helical propensity. Helix 1 is also the region with the highest α-helical propensity at pH 2.5 but its predicted α-helical propensity is two fold lower.

### Conformational Properties of Synthetic URN1-FF Peptides

To confirm the above conformational and aggregation predictions we designed five peptides encompassing the entire URN1-FF amino acid sequence (Figure 7G). The N_t_ peptide corresponds to residues 1–7 at the N-terminus, which are devoid of any regular secondary structure in the native protein. The H1 peptide corresponds to the complete helix 1 (residues 8–20). The H2 peptide comprises the stretch 21–35 including loop 1 and helix 2. The 3_10_ peptide includes loop 2 and the 3_10_ helix. These residues (36–45) connect the helices 2 and 3 in the native structure. Finally, the H3 peptide includes residues 46–59 at the C-terminal helix 3. All peptides were prepared with both termini unprotected.

**Figure 8:**
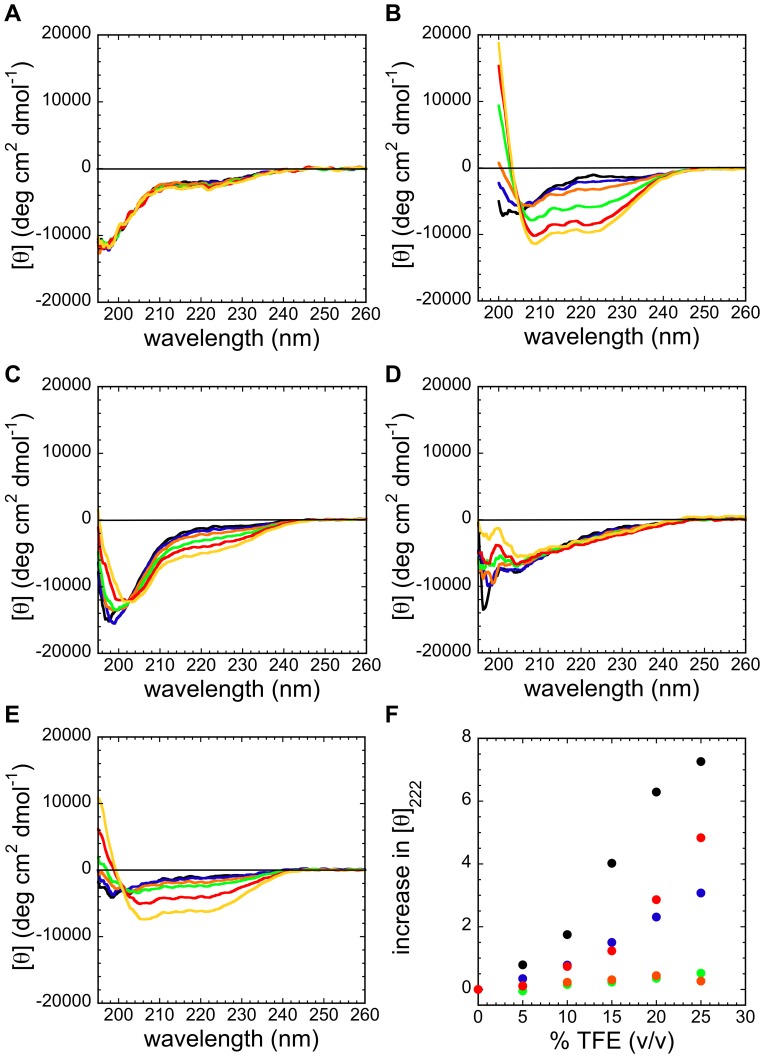
α-helical structure of synthetic URN1-FF peptides. Synthetic peptides were prepared at 100 µM and pH 2.5. Their far-UV CD spectra were recorded at 298 K in the absence (black) and in the presence of different percentages (v/v) of TFE: 5% (v/v) (blue); 10% (v/v) (orange); 15% (v/v) (green); 20% (v/v) (red); and 25% (v/v) (yellow). (**a**) N_t_, (**b**) H1, (**c**) H2, (**d**) 3_10_ and (**e**) H3 peptides. (**f**) Increase in the molar ellipticity at 222 nm versus the TFE concentration for all the synthetic peptides: N_t_ (green); H1 (black); H2 (blue); 3_10_ (orange) and H3 (red) peptides. The *Y axis* corresponds to the difference between each value and the value at 0% of TFE, divided by the value in the absence of TFE for each peptide.

We first assessed the conformational properties of the URN1-FF peptides using far-UV CD at 100 µM, in 100 mM glycine buffer at pH 2.5 and 298 K (Figure 8A–E). In this condition all the peptides exhibited an essentially unstructured conformation with minima below 200 nm, in agreement with the general observation that α-helices are only slightly stable in most short peptides in aqueous solution [38], [39]. Trifluoroethanol (TFE) has been recurrently used to stabilize the α-helical structure in short peptides [40], [41], [42]. Several proteins have been split into peptide fragments, which showed a tendency to form α-helices in TFE, even if they were unstructured in water. This propensity is particularly strong for peptides corresponding to α-helical regions in the intact protein [43].

Therefore, we tested the effect of TFE in the 5–25% (v/v) concentration range on the CD spectra of URN1-FF peptides at pH 2.5 (Figure 8A–E). In agreement with the poor secondary structure content of the correspondent protein regions in the native state, the N_t_ and 3_10_ peptides did not show evidence of secondary structure formation in any solvent condition. The H1 and H3 peptides exhibited a shift of the molar ellipticity minima toward 210 at 222 nm at increasing TFE concentrations indicating the adoption of an α-helical conformation, consistent with these two segments being the largest α-helical regions in the native URN1-FF structure. The molar ellipticity at 222 nm also increases with TFE concentration in the H2 peptide, despite the signal at 210 nm is not evident, indicating a certain propensity to populate the α-helical state but clearly lower than in the case of the H1 and H3 peptides. This is consistent with a smaller length of helix 2 in the native state.

The plot of the increase in molar ellipticity at 222 nm versus TFE concentration allows comparing the effect of the solvent on the gain of α-helical conformation in the different peptides (Figure 8F). The TFE effect is higher for H1, followed by H3 and then H2, with no apparent structural induction in the N_t_ and 3_10_ peptides. These results are in agreement with the highest α-helical propensity predicted by AGADIR for helix 1 at pH 2.5.

### Aggregation Properties of Synthetic URN1-FF Peptides

**Figure 9:**
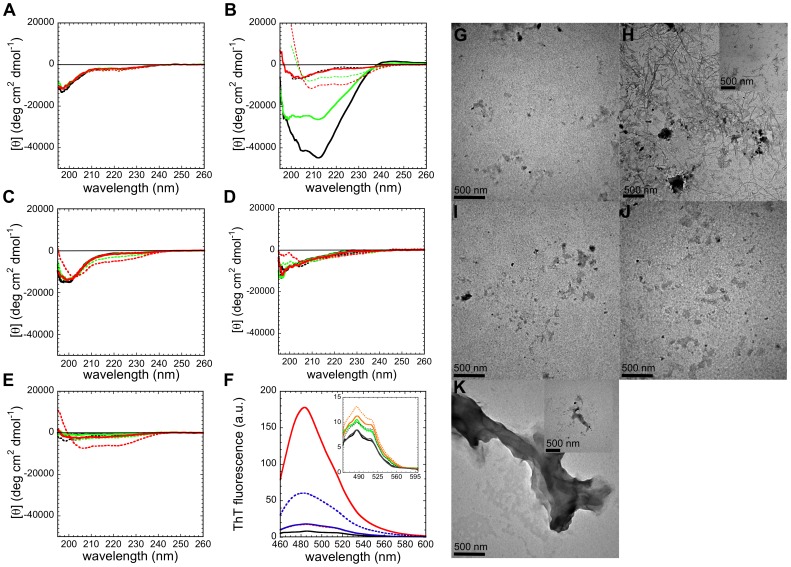
Aggregation properties of synthetic URN1-FF peptides. (**a–e**) URN1-FF peptides were prepared at 500 µM, pH 2.5, 310 K in the absence and in the presence of TFE. Protein samples incubated for one week were diluted to a final concentration of 100 µM and their far-UV CD spectra (solid lines) were compared with those of peptides under non-aggregating conditions (dashed lines). (**a**) N_t_, (**b**) H1, (**c**) H2, (**d**) 3_10_ and (**e**) H3 peptides in 0% (black), 15% (green) and 25% (red) (v/v) TFE. (**f**) Fluorescence emission spectra of ThT (25 µM) in the absence (black solid line) and in the presence of 40 µM aggregated H1 (red) and H3 (blue) peptides. In the inset, we show aggregated N_t_ (green), H2 (orange) and 3_10_ (grey) peptides. In all cases, samples in the absence of TFE are represented as solid lines, and samples with 25% (v/v) TFE are indicated as dashed lines. (**g–k**) TEM images of synthetic peptides are shown in the absence of TFE: (**g**) N_t_, (**h**) H1, (**i**) H2, (**j**) 3_10_ and (**k**) H3. The insets show the aggregated peptides in the presence of 25% (v/v) TFE.

As a next step, we analyzed the properties of the URN1-FF peptides upon incubation under aggregation-promoting conditions (500 µM, in 100 mM glycine buffer, pH 2.5 and 310 K) in the absence and in the presence of 15% and 25% (v/v) of TFE using far-UV CD and compared them with those exhibited by the peptides under non-aggregating conditions (100 µM, 100 mM glycine buffer, pH 2.5 and 298 K) (Figure 9A–E). A negative peak at ∼217 nm, indicative of β-sheet structure, was observed in the CD spectrum of the H1 peptide in the absence of TFE (Figure 9B). The β-sheet signature is still evident in the presence of 15% (v/v) TFE. In contrast, when the H1 peptide was incubated in the presence of 25% (v/v) TFE it adopted a random coil conformation and the spectrum overlapped with the one of the peptide under non-aggregating conditions in the absence of the alcohol. We monitored the morphology and amyloid-like features of the peptide aggregates by ThT fluorescence (Figure 9F) and TEM binding (Figure 9G–K) in the absence and presence of 25% (v/v) TFE. In the absence of TFE, fibrillar structures and a strong binding to ThT were observed, compatible with an amyloid-like structure (Figure 9F and 9H). The presence of TFE reduced drastically the formation of aggregates and abolished ThT binding.

The H3 peptide exhibited a significant loss of the far-UV CD signal under aggregating conditions in the absence of TFE relative to that observed under non-aggregating conditions (Figure 9E), exhibited low binding to ThT (Figure 9F) and formed amorphous aggregates as imaged by TEM (Figure 9K), In contrast to H1, the presence of 25% (v/v) TFE promoted the appearance of a β-sheet signal in the CD spectrum of H3, an increase in ThT fluorescence, and the formation of smaller peptide aggregates, as observed by TEM.

The N_t_, H2 and 3_10_ peptides exhibited predominantly random coil structure under aggregating conditions in the absence of TFE (Figure 9A, 9C and 9D). These three peptides were insensitive to the presence of TFE: small changes were observed in their far-UV CD spectra, little aggregation was visualized by TEM and no significant binding to ThT was observed, indicating that they remain essentially soluble even at high peptide concentration and low pH.

### Accessibility of α-helices in the URN1-FF Domain at Low pH

**Figure 10:**
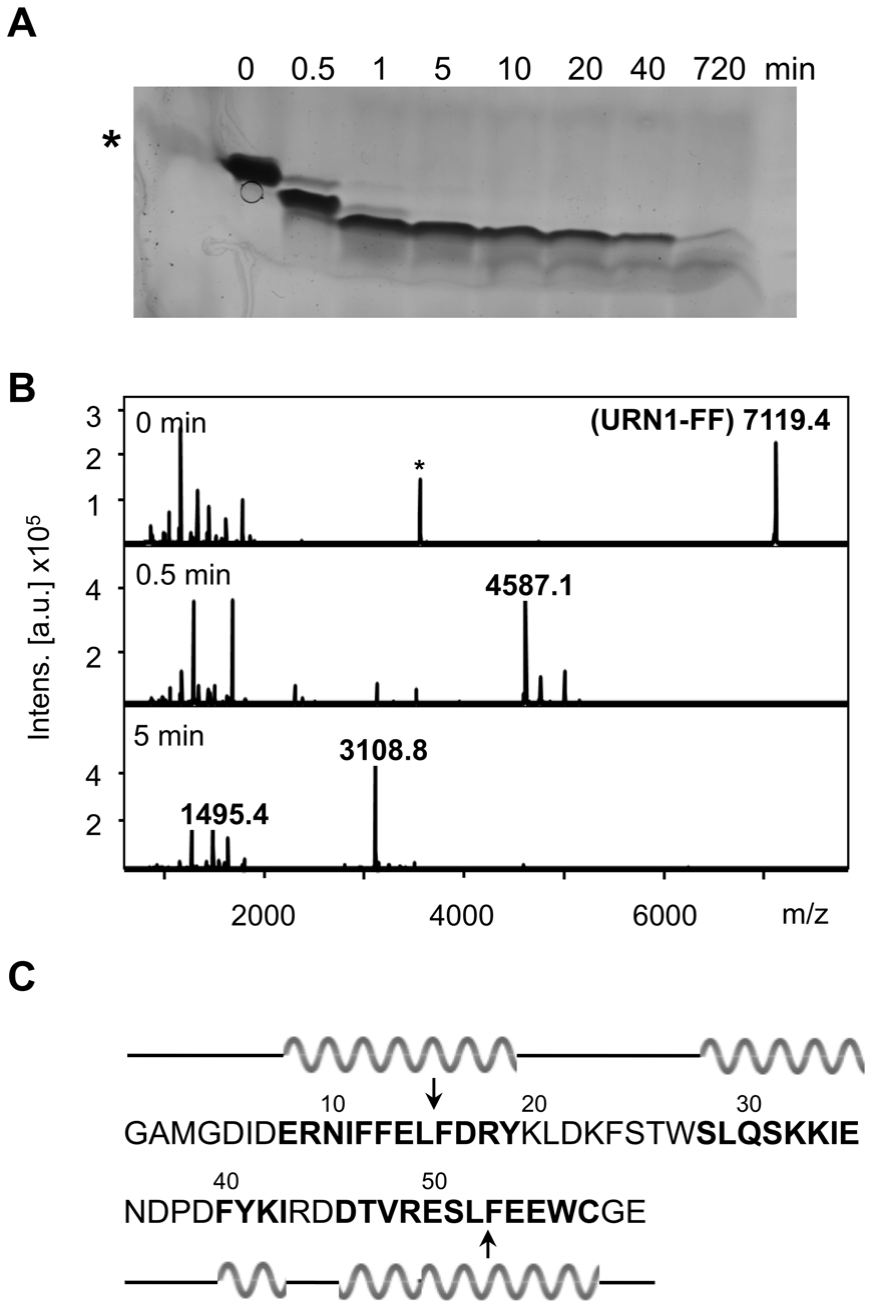
Limited proteolysis of URN1-FF at low pH. Pepsin digestion was carried out at 310 K in 50 mM glycine, pH 2.5, and 35 µM URN1-FF, using an E/S ratio of 1∶200 (by weight). (**a**) Time course proteolysis monitored by Tricine-SDS/12% (w/v) PAGE gel with Comassie blue staining. The star indicates aprotinin (6.5 kDa). (**b**) MALDI-TOFF MS before 0.5 min and 5 min after pepsin digestion. The peptides observed with a statistically significant change in abundance are denoted with their molecular weight. The star indicates a 3560 Da peak, corresponding to half URN1-FF mass. (**c**) Amino acid sequence of URN1-FF domain. The arrows indicate the pepsin cleavages inside the H1 (residue 16) and H3 (residue 52) segments. Residues forming α-helices are shown in bold. Secondary structure elements are represented above and below the sequence.

The analysis carried out with the five peptides suggests that helix 1 displays the highest α-helical and amyloid propensities at low pH and that marked propensities are also displayed by the H3 peptide, albeit to a lower extent. We used limited proteolysis to test if these two α-helices display detectable flexibility at 140 µM, 100 mM glycine buffer, pH 2.5 and 310 K in the complete URN1-FF domain and can therefore be cleaved by proteases. The domain was incubated with pepsin and the progress of the digestion reaction was monitored by SDS-PAGE on Tricine gels and by MALDI-TOF mass spectrometry (MS). A major band appeared after 30 seconds of digestion (Figure 10A) with a molecular weight of 4587 Da (Figure 10B), which could be assigned to residues 16–52. After 1 minute, a main fragment of 3108 Da was detected. This fragment corresponds to residues 16–40 and remains resistant to proteolytic attack even after prolonged incubation. It results from an internal cleavage of the original 16–52 segment. Accordingly, the appearance of the 3108 Da peak in the MS spectrum is always associated with the presence of a 1495 Da peak that matches with the predicted mass of the 41–52 fragment. Therefore, the data are consistent with the first rapid cleavages taking place inside helices 1 and 3 (Figure 10C), suggesting that they display a high conformational flexibility and low protection in the monomeric protein at low pH.

## Discussion

It is now accepted that amyloid fibril formation constitutes a generic property of polypeptide chains, including globular proteins [1]. The native structure of a protein has evolved under natural selection and is sustained by specific interactions between side-chains, which determine the backbone conformation. By contrast, repetitive backbone-backbone interactions dominate amyloid fibrils, with side chains being accommodated in the most favourable disposition compatible with the cross-β structure. Nevertheless, side chain contacts are crucial determinants of the initial transition of soluble proteins towards amyloid states, modulating thus amyloid propensity [34]. Similarly, the propensity of a polypeptide to form specific secondary structures is assumed to depend largely on its primary sequence. The transition of native α-helices to β-sheets of amyloid agregates illustrates how these structural propensities might overlap and are modulated by the structural and environmental context. α-helices and β-sheets represent alternative ways of saturating all the hydrogen bond donors and acceptors in a polypeptide backbone. Therefore, fluctuations between these two types of secondary structures involve the disruption and establishment of a significant number of non-covalent interactions. Understanding the transition between these two conformations is of interest since it has been suggested to underlie aggregation of the Aβ peptide and α-synuclein in Alzheimer’s and Parkinson’s diseases, respectively [44], [45].

Destabilization of the native state, either by solution conditions or by mutations, is a usual requirement for amyloid fibril formation in globular proteins. It usually promotes the population of partially unfolded conformers that are otherwise thermodynamically or kinetically inaccessible. Mild denaturation at low pH has been used in different protein models to induce amyloid fibril formation [46], [47]. Here, we have studied the aggregation propensity of the URN1-FF domain as a function of the pH. We observed that for this all-α protein, the native state destabilization occurring below pH 3.0 results into the formation of β-sheet enriched amyloid fibrils at physiological temperature. A variety of methodologies probing the conformational properties of soluble and aggregated states of URN1-FF and its dissected fragments have been employed to gain insights into the mechanistic aspects of the process of amyloid fibril formation.

URN1-FF remained soluble at 20 µM, 298 K, in the pH range 2.0–6.5 for 24 h. Importantly, no significant population of β-sheet structures was detected under these conditions of protein concentration and temperature in this pH range. Thermal denaturation at pH 2.0–3.0 render cooperative transitions with similar Tm and traces for CD and fluorescence probes indicating that despite the different secondary and tertiary structure content in the conformational ensembles populated at the different acidic pHs all them appear to unfold without the apparent population of intermediate states. At pH 3.0 and 2.5 the protein keeps essentially the same α-helical content than the native protein; however thermal denaturation indicates that the protein is destabilized in these conditions. At pH 2.0, the α-helical content decreases slightly and the protein stability is significantly reduced. Overall, the species populated at low pH have characteristics compatible with molten globule-like conformations [48].

As in the case of other model globular proteins, solution conditions promoting the aggregation of this α-helical domain coincide with those destabilizing the native state, but still allowing the presence of a significant amount of native secondary structure, since the types of interactions sustaining both types of structures are essentially identical. As expected, the high stability of the URN1-FF native structure at pH 5.7 prevents the protein from aggregation in this condition. By contrast, the low stability of the native state at pH 2.0–2.5 results in aggregation. Fitting of the chemical denaturation data to a two-state folding model indicates that, at pH 2.0 and 310 K, ∼50% of the URN1-FF molecules are unfolded at equilibrium. At pH 2.5 the unfolded population decreases to 10%, whereas at pH 3.0 the protein is essentially in a molten-globule state with native-like secondary structure. Therefore, the population of a significant amount of unfolded species at equilibrium is a requirement for URN1-FF aggregation into β-sheet enriched aggregates.

At pH 2.5, the aggregation rate of URN1-FF is several orders of magnitude lower than the rate for unfolding, suggesting that self-association requires a significant destabilization of the protein and at least partial unfolding of the native α-helical structure, likely because the formation of ordered intermolecular hydrogen bonds between β-strands is effectively competed by URN1-FF native helices. This explains why ordered amyloid fibrils are observed at pH 2.0 and 2.5 and not at pH 3.0, where the protein is more stable.

The computational prediction of α-helical propensity carried out with AGADIR indicates that helix 1 has the highest propensity to form this secondary structure. This prediction is supported by the experimental analysis, which also shows a good propensity for helix 3, albeit lower than that of helix 1, and a small propensity for helix 2. All computational predictions of aggregation propensity also point out helix 1 as the stretch with the highest propensity to form amyloid aggregates. In addition to this stretch, AGGRESCAN and ZYGGREGATOR predict that helix 3 also has a high amyloid propensity. According to the experimental analysis carried out with far-UV CD, ThT assay and TEM, the peptide encompassing helix 1 forms amyloid fibrils, and the peptide corresponding to helix 3 features some tendency to aggregate, although it does not form *bona fide* amyloid fibrils. Overall, it appears that helix 1, and to a lower extent helix 3, have a high propensity to form both α-helical propensity and β-sheet containing aggregates.

Thus, the FF domain illustrates how nature finds difficult to avoid the presence of aggregation-prone regions in proteins because, at least in certain cases, residues leading to the formation of native structures are also able to trigger self-assembly since both processes involve the development of similar inter-residues interactions between transiently unfolded protein regions. In these cases, it is the stability of the native conformation, and more specifically the protection of the sequence stretches with the highest aggregation propensities, that prevents the transition towards amyloidogenic conformations. Accordingly, natural mutations associated with familial forms of amyloidosis have been shown to reduce the stability of the folded state [49], [50]. As illustrated here for the helix 1, stabilization of hydrogen bonds in α-helical structures, in this case by adding TFE, reduces their ability to self-assemble into β-sheets. Therefore, compounds that target α-helical structure in disease-related proteins and reduce their conformational fluctuation might become specific aggregation inhibitors. In fact, this approach has already proven successful to reduce Aβ peptide induced *in vivo* neurodegeneration [44] and has been suggested as a promising strategy to tackle α-synuclein aggregation in Parkinson’s disease, dementia with Lewy bodies and other synucleinopathies [45]. In addition, further stabilization of α-helical structure of protein segments that possess an intrinsically high propensity to form this type of secondary structure lead to significant inhibition of aggregation [51], [52]. The data presented here reinforce the view that one of the strategies used by evolution to neutralize amyloidogenic stretches of proteins is to hide them into stable α-helical structures [53], [54].

The yeast URN1-FF domain is significantly more stable that the structurally homologous HYPA/FBP11-FF domain. Kinetic analysis indicates that URN1-FF also displays a much lower unfolding rate than HYPA/FBP11-FF. Because both thermodynamic and kinetic stabilities are important contributors to the aggregation propensity of globular proteins [55], it would be very interesting to address the aggregational properties of the HYPA/FBP11-FF domain and how they compare with that of URN1-FF. Overall, the FF domain emerges as a new and useful protein model to dissect the molecular determinants accounting for the transition between soluble and aggregated protein states.

## Supporting Information

Figure S1 **Evolution of the conformational properties of soluble URN1-FF species with time.** Protein samples were prepared at low protein concentration (20****µM), at 298, K and at pH ranging from 1.5 to 6.5. **(a)** Total tryptophan intrinsic fluorescence was measured after 1.5 h (triangles), 6 h (squares) and 24 h (circles) of sample preparation. **(b)** Far-UV CD signals at 230 nm (empty symbols) and 215 nm (filled symbols) were recorded after 3 h (triangles) and 24 h (circles) of protein dissolution. **(c)** Light scattering was followed at 350 nm after 3 h (triangles) and 24 h (circles) of sample preparation. The low scattering and CD signals at pH 4.0 result from the fact that a fraction of the protein is isoelectrically precipitated at the bottom of the tube, a phenomenon not observed at any other pH.
(TIF)

Figure S2 **Conformational properties of URN1-FF aggregates at pH 4.0. (a)** Representative TEM image of URN1-FF aggregate at 140 µM, pH 4.0 incubated at 310 K for one week. **(b)** Fluorescence emission spectra of ANS (25 µM) collected in the absence (dotted line) and presence of 10 µM of protein aggregates (crosses).
(TIF)

Table S1 **Aggregation kinetics of URN1-FF at pH 2.5.**
(DOC)
