# Supplementary figures and images for: The N-terminal Helix Controls the Transition between the Soluble and Amyloid States of an FF Domain
Source: PLoS One. 2013 Mar 7;8(3):e58297. doi: 10.1371/journal.pone.0058297 (PMC3591442; doi:10.1371/journal.pone.0058297)

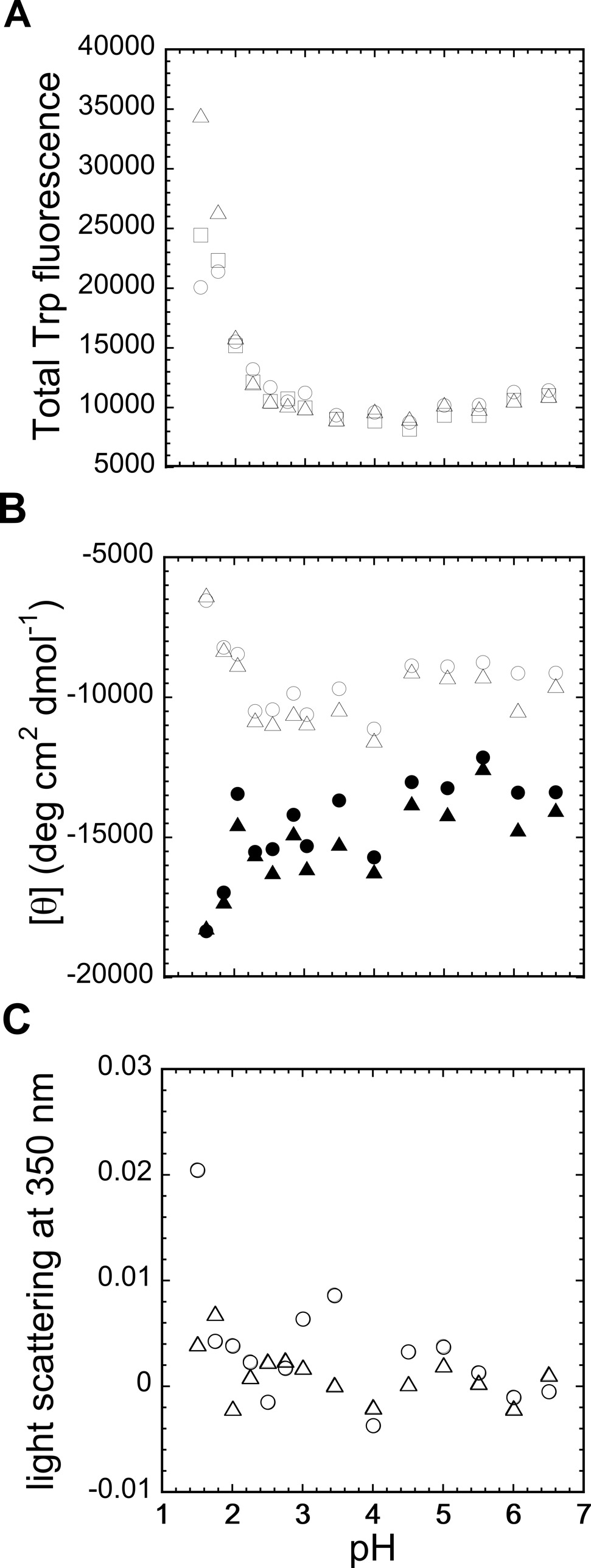

Supplement: Figure S1 — Evolution of the conformational properties of soluble URN1-FF species with time. Protein samples were prepared at low protein concentration (20µM), at 298, K and at pH ranging from 1.5 to 6.5. (a) Total tryptophan intrinsic fluorescence was measured after 1.5 h (triangles), 6 h (squares) and 24 h (circles) of sample preparation. (b) Far-UV CD signals at 230 nm (empty symbols) and 215 nm (filled symbols) were recorded after 3 h (triangles) and 24 h (circles) of protein dissolution. (c) Light scattering was followed at 350 nm after 3 h (triangles) and 24 h (circles) of sample preparation. The low scattering and CD signals at pH 4.0 result from the fact that a fraction of the protein is isoelectrically precipitated at the bottom of the tube, a phenomenon not observed at any other pH. (TIF) [file pone.0058297.s001.tif]

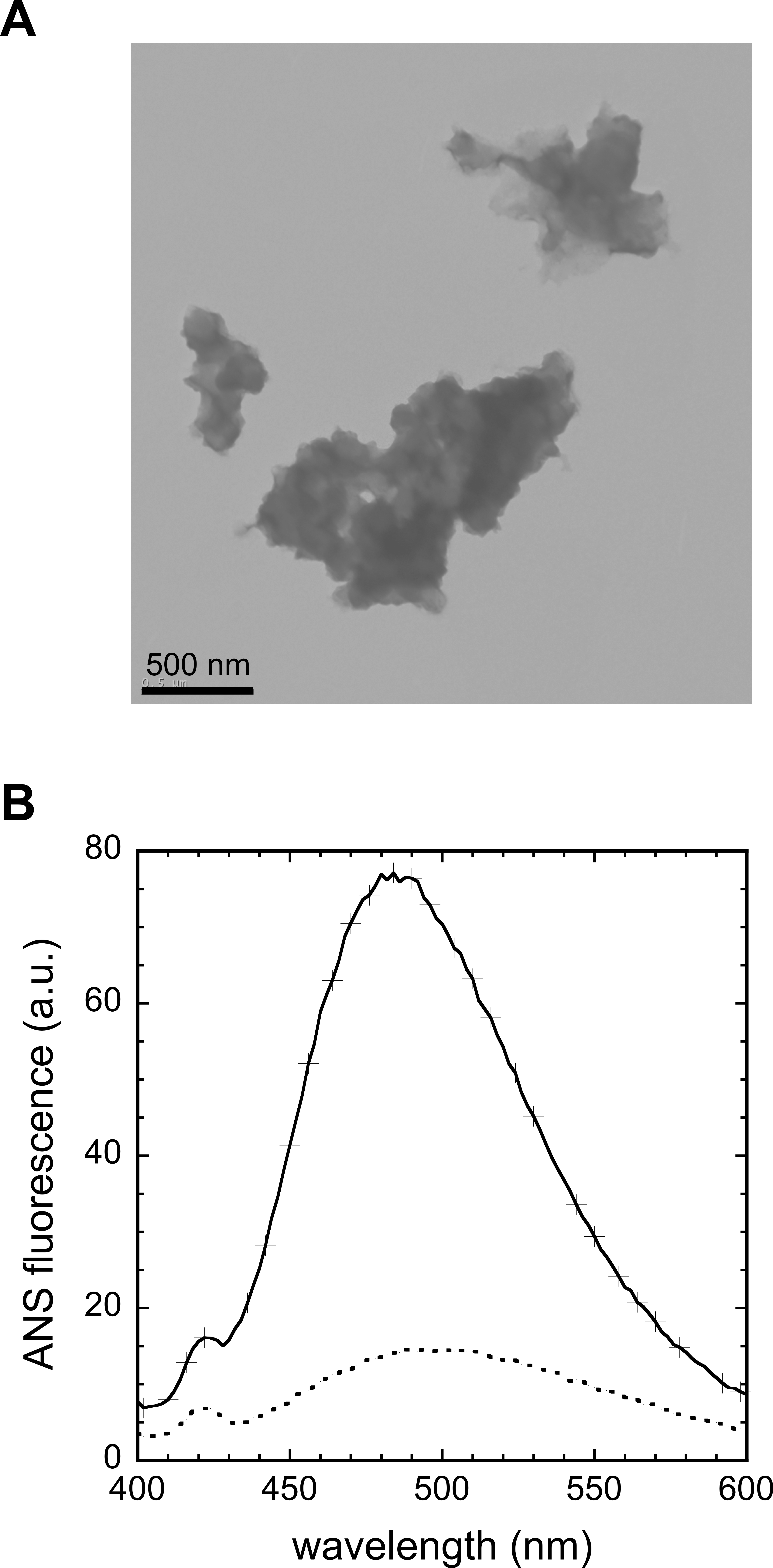

Supplement: Figure S2 — Conformational properties of URN1-FF aggregates at pH 4.0. (a) Representative TEM image of URN1-FF aggregate at 140 µM, pH 4.0 incubated at 310 K for one week. (b) Fluorescence emission spectra of ANS (25 µM) collected in the absence (dotted line) and presence of 10 µM of protein aggregates (crosses). (TIF) [file pone.0058297.s002.tif]
