# Supplementary material for: The N-terminal Helix Controls the Transition between the Soluble and Amyloid States of an FF Domain
Source: PLoS One. 2013 Mar 7;8(3):e58297. doi: 10.1371/journal.pone.0058297 (PMC3591442; doi:10.1371/journal.pone.0058297)
Supplement: Table S1 — Aggregation kinetics of URN1-FF at pH 2.5. (DOC) [file pone.0058297.s003.doc]

**Table S1. Aggregation kinetics of URN1-FF at pH 2.5**

|  | **Lag time (min)** | **k_e_ (min^-1^)** |
| --- | --- | --- |
| Thioflavin-T | 31 | 0.0077±0.0004 |
| Scattering | 34 | 0.0102±0.0009 |
